# Significance and stability of deep learning-based identification of subtypes within major psychiatric disorders

**DOI:** 10.1038/s41380-022-01482-1

**Published:** 2022-02-28

**Authors:** Nils R. Winter, Tim Hahn

**Affiliations:** Institute for Translational Psychiatry, University of Münster, Münster, Germany

**Keywords:** Psychiatric disorders, Diagnostic markers

## To the Editor:

In a recently published report, Chang et al. aim at identifying biological subtypes of major psychiatric disorders based on resting-state functional magnetic resonance imaging (rsfMRI) [1]. Surprisingly, the authors do not report any measure of significance, stability, or generalizability of their cluster solution, neither using within-sample methods nor using an independent hold-out sample. In this correspondence letter, we will describe three fundamental problems arising from the reported analytical procedure, i.e., the generalizability of the autoencoder representation, the statistical significance as well as the assignment stability of the clustering solution.

With the advancement of rich datasets and unsupervised machine learning algorithms, identifying neurobiological subtypes of psychiatric disorders has become one of the major routes promoted by biological psychiatry to establish a biologically meaningful foundation for common disorders [2, 3]. For example, Drysdale et al. have proposed four distinct biotypes of depression based on resting-state fMRI data [4]. However, in an excellent response, Dinga et al. clearly demonstrate the instability, missing significance as well as non-replicability of the reported biotypes [5]. Unfortunately, similar criticism applies to the deep learning-based clustering described by Chang and colleagues.

Chang et al. apply a feature selection based on linear models to select brain voxels that significantly differentiate between healthy controls and patients. This reduced subset of features serves as input to the autoencoder neural network to learn an even lower-dimensional representation of the data. The authors correctly mention that autoencoder neural networks can learn non-linear relationships in high-dimensional data. However, this increased capacity comes at a cost: a higher likelihood of overfitting to the training data. The amount of overfitting in small samples is generally estimated through cross-validation [6]. As the authors did not provide any cross-validation of the autoencoder model, the reader cannot judge the generalization of the learnt data representation. To make matters worse, although this feature selection and dimensionality reduction step was applied to the resting-state data only, the following statistical tests to differentiate between derived psychiatric subtypes in other modalities might be biased due to the circularity of the analysis, providing a potential danger of double dipping [7, 8].

An often-raised issue in cluster analysis is the assessment of the statistical significance of the cluster solution, i.e., assuring that the found clusters do exist and are not merely a result of random artifacts in the data. A variety of different measures have been proposed to assess the significance of a cluster solution [9]. Importantly, Chang et al. only report an external validation by running statistical tests on additional data modalities to differentiate between the two learnt psychiatric subtypes. This, however, does not provide sufficient evidence that the subtypes do exist. In addition, Chang et al. report a novel robustness measure for their cluster solution that assesses the similarity between multiple runs of the clustering algorithm, yet without providing any validation of this procedure. This is not a valid statistical test to ensure that a cluster structure exists. Similar robustness scores might occur even in data that does not contain an underlying cluster structure.

Even if the statistical significance indicates the existence of clusters, this does not necessarily mean the derived cluster solution is reliable and stable. To make sure the clusters can be interpreted reliably, it is important to evaluate the stability of the clustering assignment. Such a procedure assesses how consistently patients are assigned to a psychiatric subtype even under small perturbations of the data. Yet, an assessment of the stability of the cluster assignment is missing altogether. The previously mentioned robustness score does not provide sufficient information on the stability of the cluster solution.

**Fig. 1:**
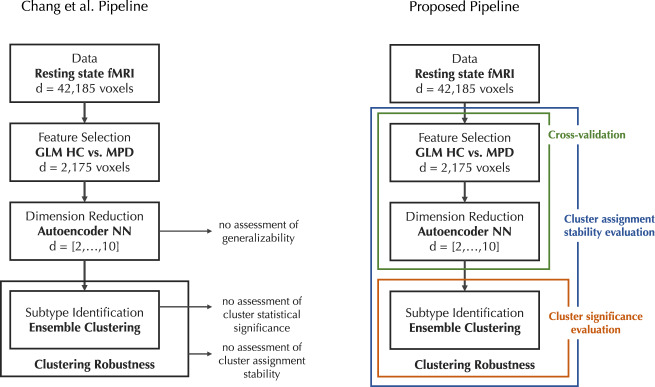
Visual representation of the analysis design employed by Chang et al. A suggested alternative pipeline is provided on the right and contains methods for assessing the generalizability, the significance, and the stability of the cluster solution. This figure was adapted from Dinga et al. [5].

Fortunately, a remedy is simple (see Fig. 1) [5]. First, to assess the generalizability of the autoencoder data representation, a cross-validation framework should be used dividing the sample into training and test set, also incorporating the initial feature selection step. Second, to guarantee that the reported cluster solution does not occur from any data with no inherent cluster structure, the procedure outlined by Liu et al. should be used to generate an empirical null distribution of a cluster criterion and the novel robustness measure [9]. This will indicate how likely it is to get a robustness score of over 0.8 with a two-cluster solution from a single multivariate Gaussian distribution. Third, in addition to cluster significance, the stability of the cluster assignment should be evaluated using a leave-one-out jack-knife procedure. For that, the complete analysis including feature selection, autoencoder training and clustering is repeated n times while leaving out one subject in every run, as has been described in detail by Dinga et al. [5]. This small perturbation of the data can subsequently be used to assess whether subjects can be reliably assigned to one of the two proposed psychiatric subtypes. Finally, to make sure that the final statistical tests comparing cluster group means in additional data modalities are not biased by the feature selection and clustering procedure due to circularity, a permutation approach can be used to generate empirical null distributions for all subsequent group difference tests [7]. This will ensure an unbiased estimate of the statistical significance of the psychiatric subtype differences.
